# Short-term survival in extensive craniofacial resections

**DOI:** 10.6061/clinics/2021/e2836

**Published:** 2021-05-11

**Authors:** Ana Kober N. Leite, Gustavo Fernandes de Alvarenga, Sérgio Gonçalves, Alexandre Bezerra dos Santos, Hugo Sterman, Claudio R. Cernea, Marco Aurélio V. Kulcsar, Luiz Paulo Kowalski, Leandro Luongo Matos

**Affiliations:** I Departamento de Cirurgia de Cabeca e Pescoco, Instituto do Cancer do Estado de Sao Paulo (ICESP), Hospital das Clinicas HCFMUSP, Faculdade de Medicina da Universidade de Sao Paulo, Sao Paulo, SP, BR; II Departamento de Neurocirurgia, Instituto do Cancer do Estado de Sao Paulo (ICESP), Hospital das Clinicas HCFMUSP, Faculdade de Medicina da Universidade de Sao Paulo, Sao Paulo, SP, BR

**Keywords:** Craniofacial Surgery, Surgery, Survival, Prognosis, Complications

## Abstract

**OBJECTIVES:**

Craniofacial resection (CFR) procedures for craniofacial tumors with cranial extension are often extensive. Although CFRs may yield good oncological results, there are concerns about high perioperative morbidity and mortality. This study aimed to determine risk factors for perioperative mortality after open CFR in terms of deaths occurring during index hospitalizations.

**METHODS:**

We conducted a retrospective analysis of CFRs conducted at a tertiary oncology hospital from May 2009 through December 2018.

**RESULTS:**

Our analysis included data from the medical records of 102 patients, the majority of whom were male (n=74, 72.5%). The mean age was 61 years (±18.3 years). Skin malignancies (n=64, 63.4%) accounted for nearly two-thirds of the treated tumors, and most of these were squamous cell carcinoma. Postoperative medical complications occurred in 33 patients (33%), and surgical complications occurred in 48 (47%). Multivariate analysis revealed the only independent risk factors for perioperative deaths to be the presence of intracranial tumor extension on preoperative imaging (hazard ratio [HR]=4.56; 95% confidence interval [CI]: 1.74-11.97; *p*=0.002) and the unexpected emergence of postoperative neurological dysfunction (HR=10.9; 95% CI: 2.21-54.3; *p*=0.003).

**CONCLUSIONS:**

In our study, factors related to tumor extension were associated with a higher risk of perioperative death.

## INTRODUCTION

Craniofacial resections (CFRs) are complex procedures that have evolved, particularly in terms of safety and expanded indications, since their first description (1). Advances in imaging, surgical technology, and reconstruction techniques have redefined the applications and limits of CFRs, with wider resections coinciding with encouraging outcomes (2,3). However, some published series describe increased overall complication rates up to 65% (2,4-6). Even though CFR is the treatment of choice for a wide variety of benign and malignant lesions involving the skull base, these surgeries are relatively uncommon, except in high-volume, specialized centers. This infrequency—in combination with differences in tumor histology and varying definitions of CFRs—leads to heterogeneous reporting in the literature (3,7,8).

Several studies have demonstrated low perioperative death rates, up to 7.6%, associated with CFRs for craniofacial tumor treatment (3-6,9-11). However, the definition of perioperative mortality varies widely in the literature, and some related publications do not describe the criteria used to define the perioperative period (12). Few reports focus on the perioperative period, and little data are available regarding factors associated with short-term postoperative survival.

An international collaborative study was conducted to overcome the difficulty inherent in obtaining large numbers at a single institution, and this culminated in the largest (N=1193) published series analyzing perioperative mortality following CFR. The resulting publication, one of the few to analyze perioperative mortality, reports the presence of at least one associated medical comorbidity (affecting 14.2% of patients; hazard ratio [HR]=1.9) as the only independent predictor of perioperative mortality (5). However substantial, these findings are fraught by the limitations of multi-institutional and retrospective studies, particularly in terms of the heterogeneity among patients and medical teams, data collection, and missing data.

This study aimed to describe the short-term outcomes of patients who underwent extensive CFR at a single tertiary cancer treatment institution and to identify the risk factors for perioperative death.

## PATIENTS AND METHODS

The data of patients who underwent CFR at Instituto do Câncer do Estado de São Paulo (ICESP, São Paulo, Brazil) between May 2009 and December 2018 were retrospectively analyzed. All included patients underwent open resections, either with combined transcranial, transfacial approaches for tumors involving the skull base or with skull-convexity resections for scalp tumors. All resections were performed by teams composed of head and neck surgeons and neurosurgeons. Microsurgical free flaps were performed by plastic surgeons.

The study was approved by the institutional review board of Hospital das Clínicas da Faculdade de Medicina da USP (number 228/14, CAAE 32884214.5.0000.0065).

The medical charts were retrospectively reviewed, and clinical, histopathological, surgical, and perioperative data were retrieved and analyzed. Perioperative deaths were those that occurred during the hospital admissions coinciding with the respective index CFR operations; this definition was not bound by time parameters. Intracranial tumor extension was defined as bulky tumor extension beyond the dural border.

The American Society of Anesthesiologists (ASA) Physical Status Classification System was used for preoperative evaluations (13).

Surgical complications were classified as surgical site complications (wound dehiscence, wound infection, flap loss, and cerebrospinal fluid fistula, for example) and medical complications (pneumonia, myocardial infarction, urinary tract infection, stroke, and thrombosis, for example).

Since CFRs can be performed to treat a variety of tumors from different locations and since the primary outcome of interest in this study was perioperative mortality, we opted to categorize outcomes according to the type of procedure (skull convexity or skull base) and not according to the tumor location. These comparisons are interdependent, and the procedure type is more relevant when evaluating postoperative complications and mortality.

Categorical data are described as absolute (n) and relative frequencies (percentage), and quantitative data are expressed as means and standard deviations. Cox regression models were used for both univariate (enter method) and multivariate analyses (with the backward likelihood ratio method, which involves removal testing based on the probability of the likelihood-ratio statistic based on the maximum partial likelihood estimates) to calculate HRs with 95% confidence intervals (CIs). Only the variables with *p*<0.20 in the univariate analysis were included in the multivariate analysis. Variables considered as dependent on others were not included in the multivariate analysis. The Kaplan-Meier method was used to estimate cumulative survival, and the log-rank test was used to compare survival curves. All analyses were performed using SPSS Statistics for Windows, version 26.0 (IBM Corp., Armonk, NY, USA), and *p*-values below 5% (*p*<0.05) were considered significant.

## RESULTS

**Figure 1:**
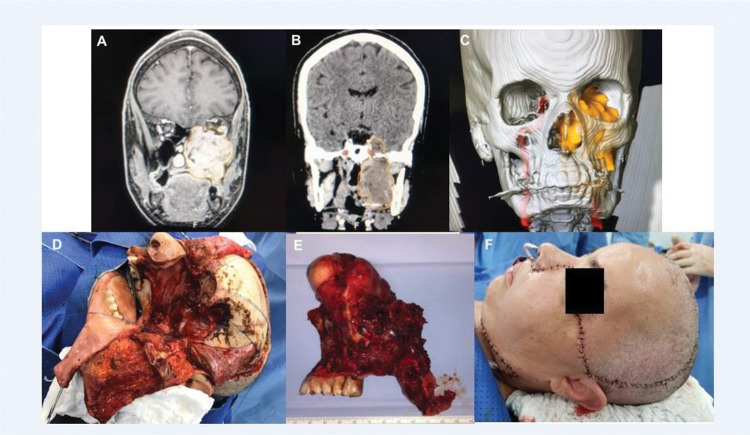
An extensive adenoid cystic carcinoma with skull base invasion submitted to craniofacial resection and microsurgical free flap reconstruction. No major complications. Images A, B, and C show preoperative scans showing a large tumor involving the maxilla and middle fossa; D, the surgical defect after *en bloc* tumor resection; E, the surgical specimen; and F, the immediate result after reconstruction.

The case series was composed of 102 patients who underwent CFR. There were 100 malignant tumors and two benign tumors. The majority of patients were male (n=74, 72.5%), and the mean age was 61 years (±18.3 years). Forty-four patients (43%) had undergone some previous cancer treatment, with surgery (n=38, 37.3%) predominating among the previous interventions; 6 patients (5.8%) had previously undergone radiotherapy. The most common tumor site of origin was the skin (n=64, 63.4%), followed by the sinonasal cavities (n=19, 18.8%) (Figure 1). The most common histologic type was squamous cell carcinoma (n=54, 52.9%), followed by basal cell carcinoma (n=12, 11.8%) and sarcomas (n=8, 7.8%). The majority of patients were smokers (n=54, 52.9%) and had associated medical comorbidities (n=63, 61.8%). The majority of patients were ASA II (n=53, 52%), followed by ASA III (n=46, 45.1%) (Table 1).

Fifty-two patients (50.9%) underwent skull-convexity resections, and 50 (49.1%) underwent skull-base resections. All patients received preoperative prophylactic antibiotics (cefuroxime), following institutional protocols. There was only one significant intraoperative complication (bleeding) reported. The mean anesthesia time was 12 hours (±245 min; range: 140-1245 min), with 68% of procedures lasting longer than 10 hours. The majority of patients underwent microsurgical free-flap reconstructions (n=72, 70.6%), which were indicated when large defects precluded local reconstruction. The mean intensive care unit admission duration was 7.3±9.6 days (range: 0-63 days), and the mean hospital stay was 17.1±14.2 days (range: 1-65 days).

Forty-eight patients (47%) had at least one surgical complication, and 34 (33.3%) had medical complications, the most common being pneumonia (17.6%) (Table 2). Nineteen patients (18.6%) died postoperatively without being discharged from the hospital; among these deaths, the longest postoperative interval was 45 days.

**Figure 2:**
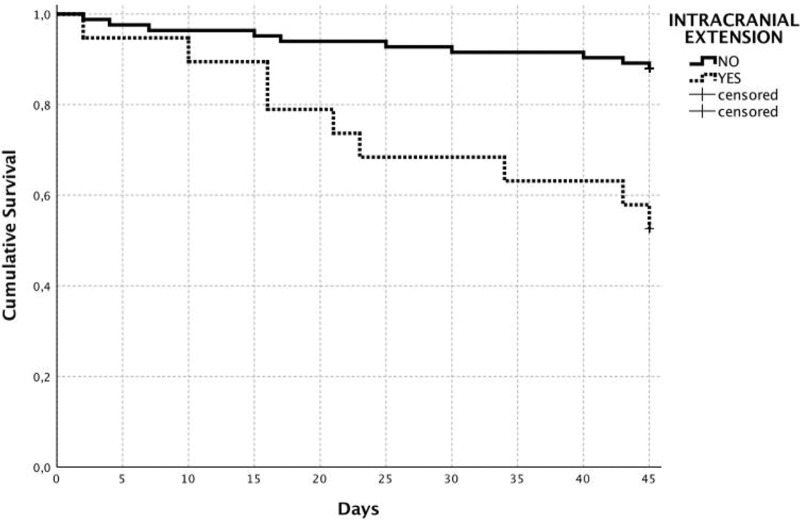
Kaplan-Meier curves demonstrating poor cumulative survival among patients with intracranial tumor extension (88.0 *vs.* 52.6%; *p*<0.001; log-rank test).

Univariate analysis determined the following variables as associated with in-hospital mortality was the presence of pneumonia (*p*=0.02), postoperative bleeding (*p*=0.01), the unexpected emergence of postoperative neurological dysfunction (*p*=0.04), microsurgical free-flap reconstruction (*p*=0.03), the need for reoperation (*p*=0.03), dural invasion on preoperative imaging (*p*=0.008), and the presence of intracranial tumor extension on preoperative imaging (*p*<0.001). According to the multivariate analysis, the only independent predictors of perioperative death were the presence of intracranial tumor extension on preoperative imaging (HR=4.56; 95% CI: 1.74-11.97; *p*=0.002) and the unexpected emergence of postoperative neurological dysfunction (HR=10.9; 95% CI: 2.21-54.3; *p*=0.003) (Table 3). Moreover, 10 of 83 patients without intracranial tumor extension (cumulative survival: 88.0%) died during the postoperative period, in contrast with nine of 19 patients (cumulative survival: 52.6%) with intracranial extension who died within this period (log-rank, *p*<0.001) (Figure 2).

Considering this important risk and the fact that it potentially influenced some surgeons to forgo operating on such patients, patients with intracranial extension on preoperative imaging (n=19) were removed for a subgroup analysis of the remaining 83 patients. According to the univariate (subgroup) analysis, the following factors were associated with earlier perioperative deaths: the presence of postoperative medical complications (HR=4.6; 95% CI: 1.3-16.5; *p*=0.01), postoperative bleeding (HR=7.0; 95% CI: 1.8-27.2; *p*=0.005), and the unexpected emergence of neurological dysfunction (HR=7.4; 95% CI: 1.5-35.4; *p*=0.012). According to the multivariate (subgroup) analysis, the only independent risk factor for perioperative death was postoperative bleeding (*p*=0.002, HR=9.1; 95% CI: 2.2-37.4). No modifiable preoperative variables were identified as associated with perioperative mortality in these analyses.

## DISCUSSION

Craniofacial resections (CFR) are complex procedures that have evolved in safety and indications since first being described (1). Advances in imaging, surgical technology, and reconstructive techniques have led to re-defined applications and limitations, with wider resections but satisfactory oncologic results (14).

CFR is used only for specific types of tumors, making it difficult to generate prospective data on this topic from a large cohort (15). Therefore, information about which patients are more likely to have unfavorable outcomes is extremely valuable to guide future therapeutic decisions.

Endoscopic surgery is an approach that has greatly evolved over the last few decades, and it has been associated with good oncological results in some series (16). However, some criteria have been described as relative contraindications to the endoscopic approach, including involvement of the nasal bones, lateral frontal sinuses, lacrimal system, orbit, anterior maxillary sinuses, dura mater, cavernous sinus, and brain parenchyma (17), all of which were frequently documented in the present series.

This report presents data and analytical findings derived from a large cohort of patients from a single oncologic institution. These patients underwent extensive open CFRs mainly for malignant lesions, and this study focused on factors associated with perioperative death rather than long-term survival.

Perioperative deaths were those that occurred during the hospital admissions that included the respective CFR operations, and the latest perioperative death occurred 45 days after surgery. In this series, the postoperative mortality rate was 18.6%, which is higher than the 0% to 7.6% mortality rates previously reported in association with CFRs (1,3,9,18,19). However, these mortality rates have been associated with varying definitions of perioperative mortality (12), and many related publications do not clearly define perioperative mortality. It is likely that the perioperative mortality rate calculated from our series is relatively high because our definition of perioperative mortality was more inclusive than the definitions used elsewhere.

Published studies investigating CFRs have been heterogeneous in terms of histologic types, surgical techniques, surgical approaches (open or endonasal), and this—along with the small sample sizes in these studies—has led to divergent results (3,7,8). For example, Donald et al. (20) described 52 skull base operations for invasive scalp cancer; among the tumors operated on, 32% only superficially invaded the periosteum at most, and these procedures, therefore, should not have been classified as skull base operations. Yang et al. (21) described a series of 126 craniofacial resections for oral and maxillofacial tumors. However, 31% of these tumors only underwent soft tissue resections; thorough bone resections and dura mater resections were performed for 24.6% and 8.7% of cases, respectively, exemplifying the discrepancies in CFR definitions. The present series only included open resections, either with combined transcranial, transfacial approaches or with skull-convexity approaches. All included procedures were extensive operations involving cranial bone resection. The mean anesthesia time was 12 hours, and the mean tumor size was 7.7 cm. The majority of our patients required microsurgical free flaps for reconstruction (70.6%) because of large defects; in previous studies, free flaps were needed only in 4% to 21.6% of cases (2,3,6,8).

The high rate of complex microsurgical free-flap reconstructions was associated with the relatively high proportion of reoperations (30.9%), which were mostly carried out to revise vascular anastomoses. Even though free-flap reconstructions and reoperations were not associated with perioperative deaths according to the multivariate analysis, their high frequencies indirectly reflect the complexity of these operations.

Brazilian demographic and socioeconomic characteristics strongly influenced the high proportion of patients with skin cancer (63.4%), many (51%) of whom underwent skull-convexity resections, meaning that this was a unique cohort. Skin cancers with cranial invasion usually reflect a long clinical course of untreated disease, and the excision of skin and soft tissue in addition to the cranial base may predispose patients to complications. One of the main factors potentially associated with the high mortality rate was the high incidence of associated medical comorbidities, which present in 61.8% of patients, which contrasted with rates of 13% to 17% reported for other series (2,5,22). The high incidence (equally affecting all subgroups of patients) of medical comorbidities could itself explain why such morbidities were not associated with perioperative death in our analyses. A hallmark international collaborative study that analyzed early mortality after CFRs was conducted by Ganly et al. (5), who identified the presence of comorbid conditions as the only independent risk factor for perioperative mortality among 1,193 patients. Notably, only 14.2% of the patients in that study had comorbid conditions, which was much lower than 61.8% identified in our study.

There was probably an association between the high prevalence of skin cancers observed in this series and the low proportion (5.9%) of patients previously treated with radiotherapy. Forty-four patients had received some kind of previous treatment, with surgical interventions predominating—a pattern commonly observed among skin cancers. Therefore, the lack of association between previous treatment and earlier death (*p*=0.52) was expected.

For the statistical analyses, we opted to use a model of survival up to 45 postoperative days (i.e., during the same hospital admission as the index operation) rather than frame mortality as a binary variable limited to 30 days. This occured because, even though the outcome of perioperative death is binary, in clinical practice, the time that elapses before the outcome event is defined might be associated with multiple factors dependent on time, and relevant (perhaps, longer-term) components of the postoperative period should not be excluded from such analyses by simply focusing on death within a predetermined cutoff time parameter. To our knowledge, no other studies have used such criteria.

The presence of intracranial extension on preoperative imaging was the main factor associated with perioperative death (HR: 4.56). Ganly et al. (5) did not identify dural invasion or brain invasion as risk factors for death; however, it is not clear if their article refers to imaging or histopathological findings. Dias et al. (9) found the extent of surgery to be the main factor associated with complications but not with mortality. Similarly, Sakashita et al. (22) found an association between dural resections and complications; however, they did not analyze perioperative deaths. Mortality outcomes may vary by the location of intracranial extension; however, 19 patients with intracranial extension were too few to allow for a sufficiently robust statistical analysis.

The determination of preoperative variables associated with perioperative death is the most valuable in clinical practice. Based on our findings, patients with intracranial tumor extension on preoperative imaging have an almost 5-fold higher risk of perioperative death than patients without intracranial extension. This should be taken into consideration when proposing surgical treatment and discussed with the patient. However, there are no satisfactory treatment alternatives for many patients who require craniofacial; therefore, intracranial tumor extension cannot be considered an absolute contraindication, and treatment decisions should proceed on a case-by-case basis. Among the 10 patients identified with preoperative intracranial tumor extension who did not die during the perioperative period, seven (70%) were alive and without evidence of disease at the most recent follow-up visit; therefore, surgery yielded good oncological control for these patients.

Advances have been made in recent years regarding systemic cancer therapies, particularly for non-melanoma skin cancers (23,24). This scenario increases the possibility of neoadjuvant options for locally advanced lesions, but more reliable data are still required. Reigneau et al. (25) investigated the use of cetuximab with or without platinum-based chemotherapy for unresectable squamous cell carcinomas among 34 patients. The tumors became resectable in 92% of patients who received combined therapy and 55.5% of patients in the monotherapy group. For basal cell carcinomas, the hedgehog pathway inhibitor vismodegib has shown good results for locally advanced tumors (26). Some authors have shown that vismodegib can prevent the need for orbital exenteration for periocular tumors (27), with complete response rates up to 67% (28,29), but this evidence is limited by the small number of cases. Targeted immunotherapy also appears to play a role in locally advanced skin cancers, but more robust supporting evidence is still needed (30). We found no reports of specific studies investigating novel neoadjuvant therapies before CFRs. 

There may be a role for neoadjuvant therapies or even definitive non-surgical therapies for tumors invading the skull, particularly for extremely high-risk patients with intracranial tumor extension. The use of such interventions with the intent of reducing intracranial extension and, consequently, the complexity of surgery and mortality risk, is a beacon of hope in a grim scenario and should be evaluated in future prospective trials.

When the patients with intracranial tumor extension were excluded from the analysis, the only independent risk factor for earlier death was perioperative bleeding, which could not be preoperatively evaluated or prevented, emphasizing the importance of the reported findings.

This study had several limitation. Its retrospective design and relatively small number sample size were important limitations, but these shortcomings were difficult to overcome due to the nature and rarity of craniofacial tumors.

## CONCLUSIONS

Extensive craniofacial resections are extremely complex procedures that can be associated with significant short-term mortality. Indications should be carefully evaluated, especially among patients with intracranial tumor extension identified by preoperative imaging, which is associated with an increased risk of perioperative death.

## AUTHOR CONTRIBUTIONS

Leite AKN was responsible for the study design, data revision, analysis of results and manuscript elaboration. Alvarenga GF was responsible for the study design, data collection and analysis of results. Gonçalves S, Santos AB and Sterman Neto H were responsible for the study design, analysis of results and manuscript revision. Cernea CR, Kulcsar MAV and Kowalski LP were responsible for the analyses of results and manuscript revision. Matos LL was responsible for the study design, statistical analyses, analysis of results and manuscript revision.

## Figures and Tables

**Table 1:** Patient and tumor characteristics.

Variable	N (%)
Age	
Mean±Standard Deviation	61±18.3
Gender	
Male	74 (72.5)
Female	28 (27.5)
Tumor location	
Skin	64 (63.4)
Sinonasal	19 (18.8)
Minor salivary glands	5 (5.0)
Parotid	1 (1.0)
Nasopharynx	1 (1.0)
Other	11 (10.9)
Intracranial extension on preoperative imaging	
No	83 (81.4)
Yes, not touching the brain parenchyma	10 (9.8)
Yes, touching the brain parenchyma	8 (7.8)
Yes, invading the brain parenchyma	1 (1)
Location of intracranial extension	
Frontal lobe	12 (63.2)
Frontal and parietal lobe	3 (15.8)
Temporal lobe	4 (21)
Previous treatment	
No	58 (56.9)
Surgery alone	38 (37.3)
Radiation therapy alone	2 (2.0)
Surgery+chemo/radiation	4 (3.9)
Tobacco use	
No	48 (47.1)
Yes	54 (52.9)
Medical comorbidities	
No	39 (38.2)
Yes	63 (61.8)
ASA classification	
I	3 (2.9)
II	53 (52)
III	46 (45.1)

ASA: American Society of Anesthesiologists Classification.

**Table 2:** Surgical procedures.

Variable	N (%)
Type of surgery	
Skull base	50 (49)
Skull convexity	52 (51)
Surgical Complications	48 (47.1)
Wound dehiscence	12 (11.8)
Bleeding	8 (7.8)
Flap loss	15 (14.7)
Wound infection	21 (20.6)
Unexpected neurological dysfunction	4 (3.9)
CSF fistula	7 (6.9)
Reoperation <30 days	40 (30.9)

CSF: cerebrospinal fluid.

**Table 3:** Cox regression analyses identifying the variables associated with perioperative death among patients who underwent extensive craniofacial resection.

	Univariate		Multivariate	
**Variable**	HR (95% CI)	*p*-value	HR (95% CI)	*p*-value
Gender	0.63 (0.25-16)	0.340		
Age	1.02 (0.9-1.0)	0.200		
Type of resection (Skull base or cranial vault)	1.8 (0.7-4.5)	0.200		
Previous treatment	0.74 (0.29-1.8)	0.520		
ASA ≥ III	1.11 (0.45-2.7)	0.810		
Tobacco use	0.82 (0.3-2.0)	0.670		
Anesthesia time >600 min	2.62 (0.7-8.9)	0.120	0.99 (0.22-4.5)	0.991
Medical comorbidities	0.79 (0.22-2.8)	0.720		
Any medical complication	*4.98 (1.8-13.1)*	*<0.001*	*	*
Pneumonia	*2.96 (1.16-7.5)*	*0.020*	2.40 (0.90-6.38)	0.079
Any surgical complication	*3.38 (1.2-9.3)*	*0.010*	*	*
Postoperative bleeding	*3.97 (1.3-11.9)*	*0.010*	1.88 (0.6-5.94)	0.280
CSF fistula	0.04 (0-81.0)	0.410		
Microsurgical free flap	8.57 (1.14-64.2)	0.037	7.12 (0.94-53.84)	0.057
Flap loss	2.09 (0.75-5.8)	0.150	*	*
Wound infection	0.99 (0.3-2.9)	0.980		
Wound dehiscence	1.49 (0.4-5.1)	0.520		
Unexpected neurological dysfunction	*4.38 (1.0-19.0)*	*0.040*	*10.9 (2.21-54.3* *)*	*0.003*
Reoperation <30 days	*2.75 (1.1-6.9)*	*0.030*	1.49 (0.6-3.98)	0.424
Dural invasion on preoperative imaging	*2.26 (1.2-4.1)*	*0.008*	*	*
Intracranial invasion on preoperative imaging	*2.04 (1.9-11.8)*	*<0.001*	*4.56 (1.74-11.97)*	*0.002*

* Variables considered as dependent on others and, therefore, not included in the multivariate analysis.

ASA, American Society of Anesthesiologists; CI, confidence interval; CSF, cerebrospinal fluid; HR, hazard ratio.
